# Fossils in Myanmar amber demonstrate the diversity of anti-predator strategies of Cretaceous holometabolan insect larvae

**DOI:** 10.1016/j.isci.2023.108621

**Published:** 2023-12-03

**Authors:** Carolin Haug, Joachim T. Haug, Gideon T. Haug, Patrick Müller, Ana Zippel, Christine Kiesmüller, Joshua Gauweiler, Marie K. Hörnig

**Affiliations:** 1 Ludwig-Maximilians-Universität München (LMU Munich), Biocenter, Großhaderner Str. 2, 82152 Planegg-Martinsried, Germany; 2 GeoBio-Center at LMU, Richard-Wagner-Str. 10, 80333 München, Germany; 3 Kreuzbergstr. 90, 66482 Zweibrücken, Germany; 4 University of Greifswald, Zoological Institute and Museum, Cytology and Evolutionary Biology, Soldmannstr. 23, 17489 Greifswald, Germany; 5 University Medical Center Rostock, Medical Biology and Electron Microscopy Center, Strempelstr. 14, 18057 Rostock, Germany

**Keywords:** Entomology, Evolutionary biology, Paleobiology

## Abstract

Holometabolan larvae are a major part of the animal biomass and an important food source for many animals. Many larvae evolved anti-predator strategies and some of these can even be recognized in fossils. A Lagerstätte known for well-preserved holometabolan larvae is the approximately 100-million-year-old Kachin amber from Myanmar. Fossils can not only allow to identify structural defensive specializations, but also lifestyle and even behavioral aspects. We review here the different defensive strategies employed by various holometabolan larvae found in Kachin amber, also reporting new cases of a leaf-mining hymenopteran caterpillar and a hangingfly caterpillar with extensive spines. This overview demonstrates that already 100 million years ago many modern strategies had already evolved in multiple lineages, but also reveals some cases of now extinct strategies. The repetitive independent evolution of similar strategies in distantly related lineages indicates that several strategies evolved convergently as a result of similar selective pressures.

## Highlights

• Review of different defensive strategies of holometabolan larvae in Kachin amber
• New cases including hymenopteran and hangingfly caterpillars were found
• Modern and now extinct strategies were present in Cretaceous in multiple lineages
• Strategies may have evolved convergently as result of similar selective pressures

## Graphical abstract

**Figure:**
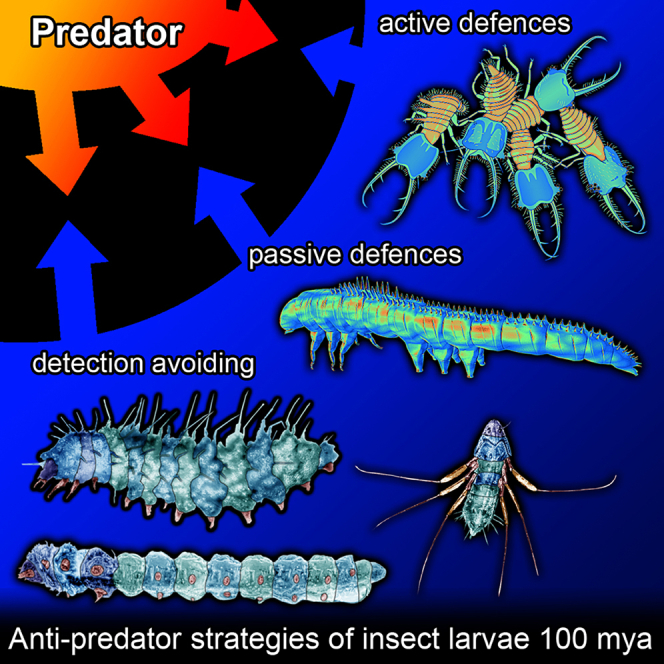

## Introduction

A large share of animal diversity and biomass in terrestrial and fresh-water ecosystems is represented by the group Insecta. More precisely, it is represented by its ingroup Holometabola with the hyperdiverse lineages of Hymenoptera (wasps), Coleoptera (beetles), Lepidoptera (moths), and Diptera (flies), as well as some less diverse groups such as Neuropterida (lacewings and allies), Mecoptera (scorpionflies), or Trichoptera (caddisflies). Holometabolans are characterized by a distinct differentiation of the early post-embryonic life stages, the larvae, which possess very different morphologies and ecologies compared to their adults. Also, quite some species spend a considerably longer time of their life span in the larval form and have only a short-lived adult phase. The larvae exhibit specializations for eating, i.e., to gather energy fast and effective,^1^ possibly representing a key feature of holometabolan larvae.

These specializations allow the larvae to transform other food sources into high-protein matter. Holometabolan larvae are therefore an interesting food source for many types of organisms, including other holometabolan larvae, but also other representatives of Insecta including adults, and not least a variety of larger organisms including mammals, lizards, and especially also many types of birds.

A strong selective pressure is therefore acting on holometabolan larvae to evolve anti-predator strategies and increase the survival rates of the larvae. Such strategies can involve, among others, different aspects of behavior, morphological features, chemical specializations, physiological changes, or also combinations of several of these factors (see also^2^). The occurrence of several anti-predator strategies can therefore also give insights into possible predator strategies, as these strategies must have evolved under certain selective pressures.

The evolutionary interaction of predator and prey is very old. We can therefore expect that anti-predator specializations of holometabolan larvae should also be around since quite some time. A look into the fossil record offers a view on this aspect. The oldest fossils of holometabolan larvae are from the Carboniferous, slightly more than 300 million years ago.^3^^,^^4^ Yet, the record in the Paleozoic is overall rather scarce.^5^^,^^6^ From the Triassic onwards, the Mesozoic has provided several larval forms,^7^^,^^8^^,^^9^^,^^10^^,^^11^ but many Lagerstätten providing a wealth of adult holometabolans have provided an astonishingly low amount of their larval forms.^12^ From the Cretaceous onward, ambers start to provide a window to the larval forms of Holometabola in the past in an almost life-like manner. While there are older ambers,^13^^,^^14^^,^^15^^,^^16^ these have so far not included holometabolan larvae. Among the different Cretaceous ambers, Kachin amber, found in Myanmar and being around 100 million years old, has been especially productive in providing examples of holometabolan larvae^17^^,^^18^ that possess different types of anti-predator structures or allow to infer anti-predator types of behavior.^19^^,^^20^^,^^21^ We here summarize the known cases of anti-predator strategies of holometabolan larvae from Kachin amber, including the report of some new findings.

The overall aim of this study is to contribute to a better understanding of the ecosystem of the Cretaceous Myanmar amber forest. For this purpose, it is necessary to understand the ecological role of a group and its possible position in the food web. With this overview, we want to examine aspects of the potential ecological importance of holometabolan larvae in the Cretaceous Myanmar amber forest, also to give a starting point for further investigations of the evolutionary change of the ecological role in deep time till today.

## Results and discussion

### Anti-predator strategies

Anti-predator strategies include all aspects which prevent or hamper a potential predator from detecting, grabbing, or killing another animal. There are several strategies directly linked to predator avoidance, such as avoidance of detection or recognition by a potential predator (camouflage, mimesis), passive defense strategies (spines and hairs, protective cases, aposematism and mimicry) or active defense strategies (group defense and escape strategies). However, there are several strategies that might not be related to defense primarily, or the main evolutionary driver cannot be clearly identified, but has defense as (side) effect. These include characters or strategies that make it more unlikely to be recognized or grasped by a predator or require further specializations of the predator. This is the case of larvae which live inside a substrate (e.g., wood, leaves, soil, or even parasitism) or further passive strategies such as unusual shape or size (however, some of these can be clearly assigned to predator avoidance, i.e., see below).

Anti-predator strategies can include complex combinations of characters and behaviors. Therefore, to understand the ecological role or the potential position of a group in a food web, one needs to consider as many influencing factors as possible. In the following, we summarize all known examples of the Cretaceous fossil record of holometabolan larvae in Myanmar amber which include strategies, direct or indirect, resulting in defense against potential predators.

### Detection avoidance and related strategies

#### Camouflaging

**Figure 1:**
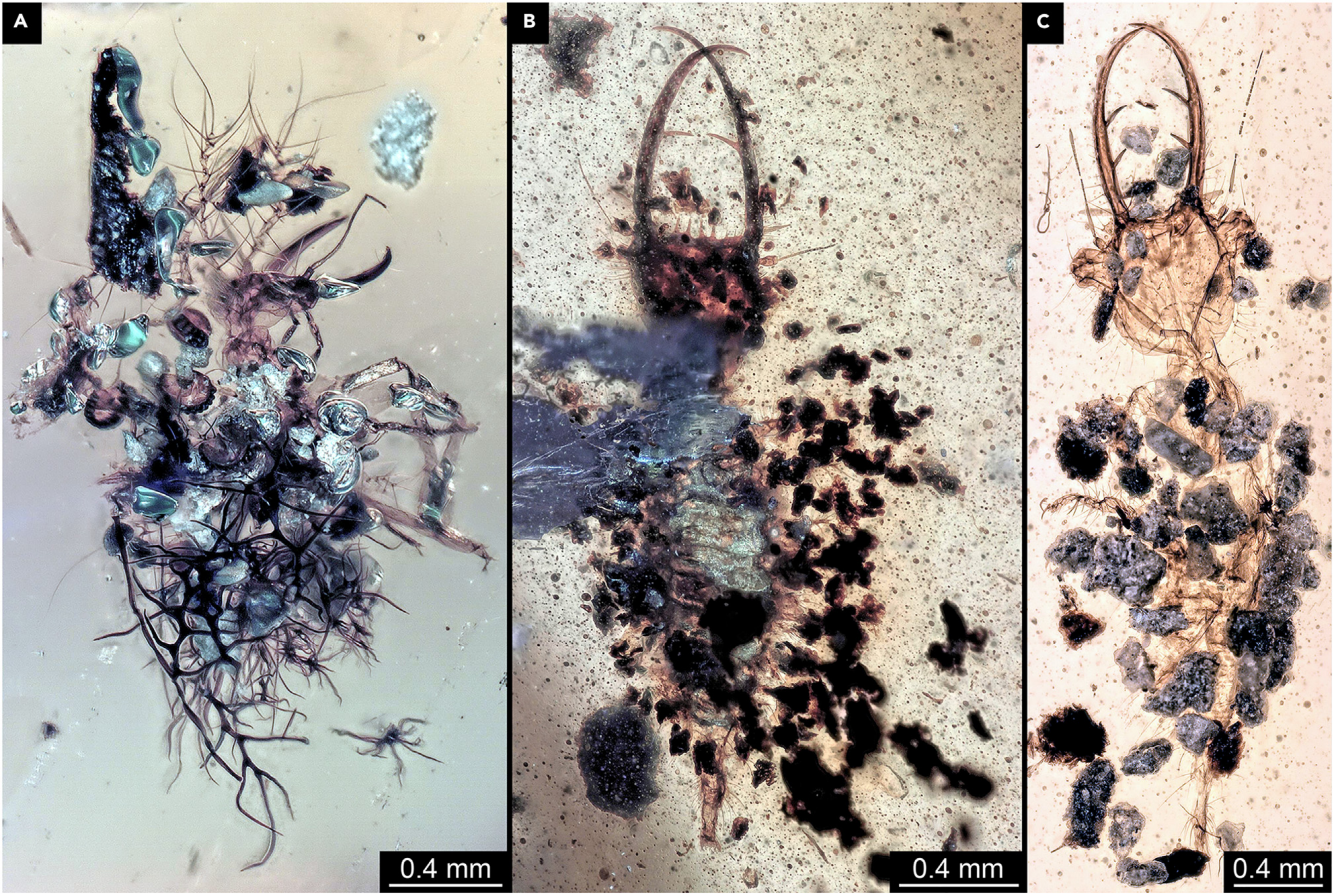
Lacewing larvae in Kachin amber with camouflaging structures (A) Aphidlion-like larva, PED 0642. (B) Larva of split-footed lacewings, PED 0377. (C) Owllion larva, BUB 3724.

An apparently common strategy also employed by non-holometabolan immatures (e.g.,^19^^,^^21^^,^^22^) is to use debris to cover larger parts of the body in order to avoid detection/recognition by the predator. Different lacewing larvae^19^ show this type of behavior in Kachin amber (and also other Cretaceous ambers^23^^,^^24^^,^^25^^,^^26^). Special structures on the dorsal side of the trunk for organizing and fixing the debris are especially developed in aphidlion-like larvae (related to modern green lacewings, Chrysopidae; Figure 1A; ^27^). Interestingly, the structures of these processes differ in detail from those known in extant aphidlions, making it most likely that such structures have evolved convergently in both cases.

In larvae of split-footed lacewings, some lateral structures are also present, but most debris is simply attached to the dorsal side (Figure 1B; ^19^^,^^28^). Similar camouflaging cloaks are also known in owllion larvae (related to the modern group Ascalaphidae or Ascalaphinae; Figure 1C; see^29^^,^^30^).

Camouflaging cloaks for some of these predatory larvae do not only allow for an optical disguise. Attaching remains of dead prey items to the cloak may also act for chemical sense camouflaging (e.g.,^31^). The camouflaging cloak in lacewings may not only be beneficial for avoiding predators (passive camouflaging), but also help in prohibiting detection/recognition by potential prey and increase hunting success. Therefore, it remains partly unclear which of the two factors had the stronger selective pressure on the animals to evolve this strategy already 100 million years ago in several lineages independently.

**Figure 2:**
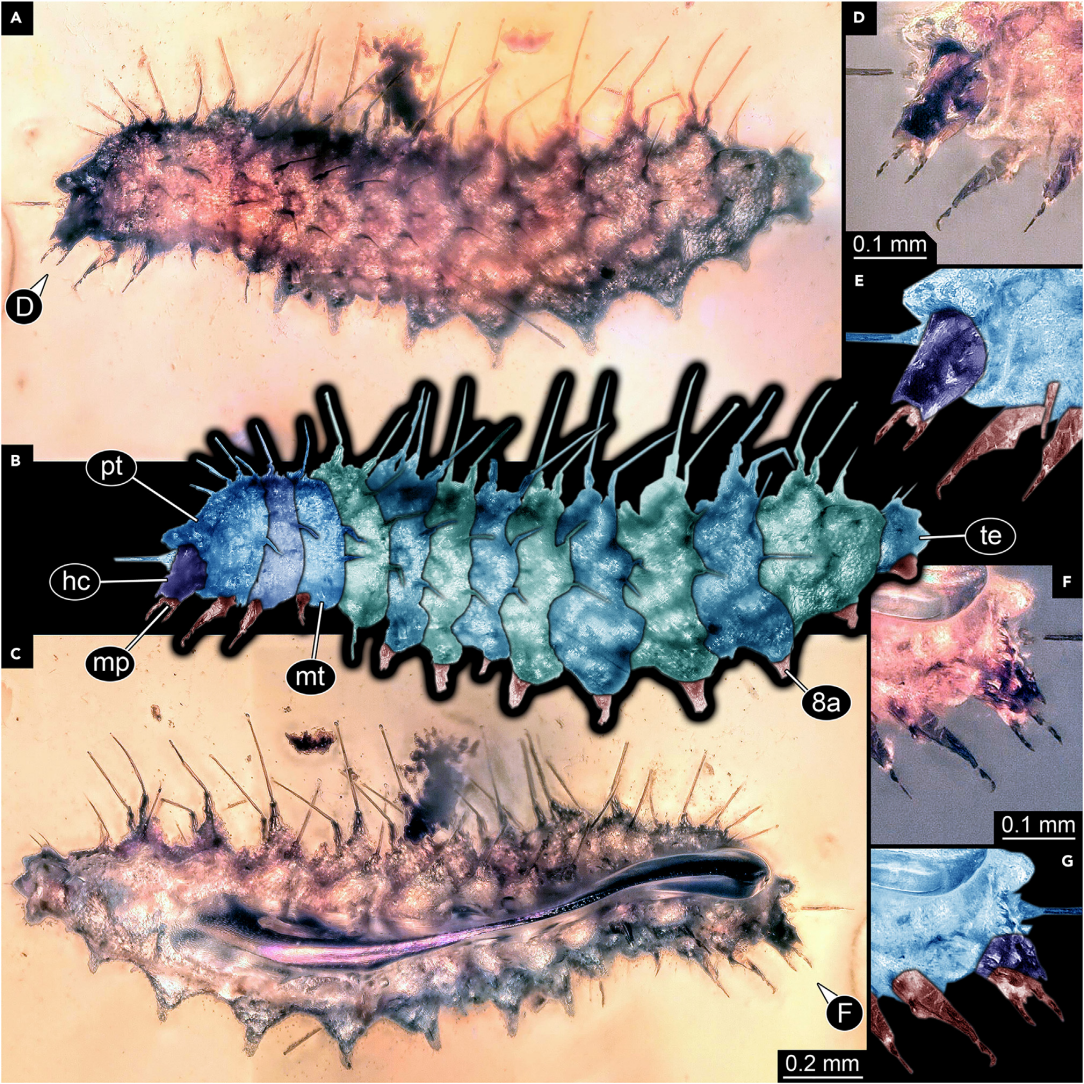
Larva of Mecoptera (Bittacidae), PED 1986 (A) Left-lateral view. (B) Color-marked version of A. (C) Right-lateral view. (D) Right-lateral close-up of the head. (E) Color-marked version of D. (F) Left-lateral close-up of the head. (G) Color-marked version of F. Abbreviations: hc = head capsule; mp = maxillary palps; mt = metathorax; pt = prothorax; te = trunk end; 8a = appendage of abdomen segment 8 (proleg).

We here report a new caterpillar, the first Cretaceous larva of the group Mecoptera (scorpionflies), more precisely most likely of the group Bittacidae (Figure 2). The dorsal side has very prominent setae of which the sockets are drawn out into long cones, just as in modern larvae of Bittacidae.^32^^,^^33^^,^^34^ These structures seem also to be used for carrying a camouflaging cloak in modern larvae,^35^ indicating that the fossil would also have employed the same strategy. The absence of such a cloak in the new fossil may be related to an early developmental state of the larva (see also discussion in^36^).

#### Mimesis (or masquerade)

**Figure 3:**
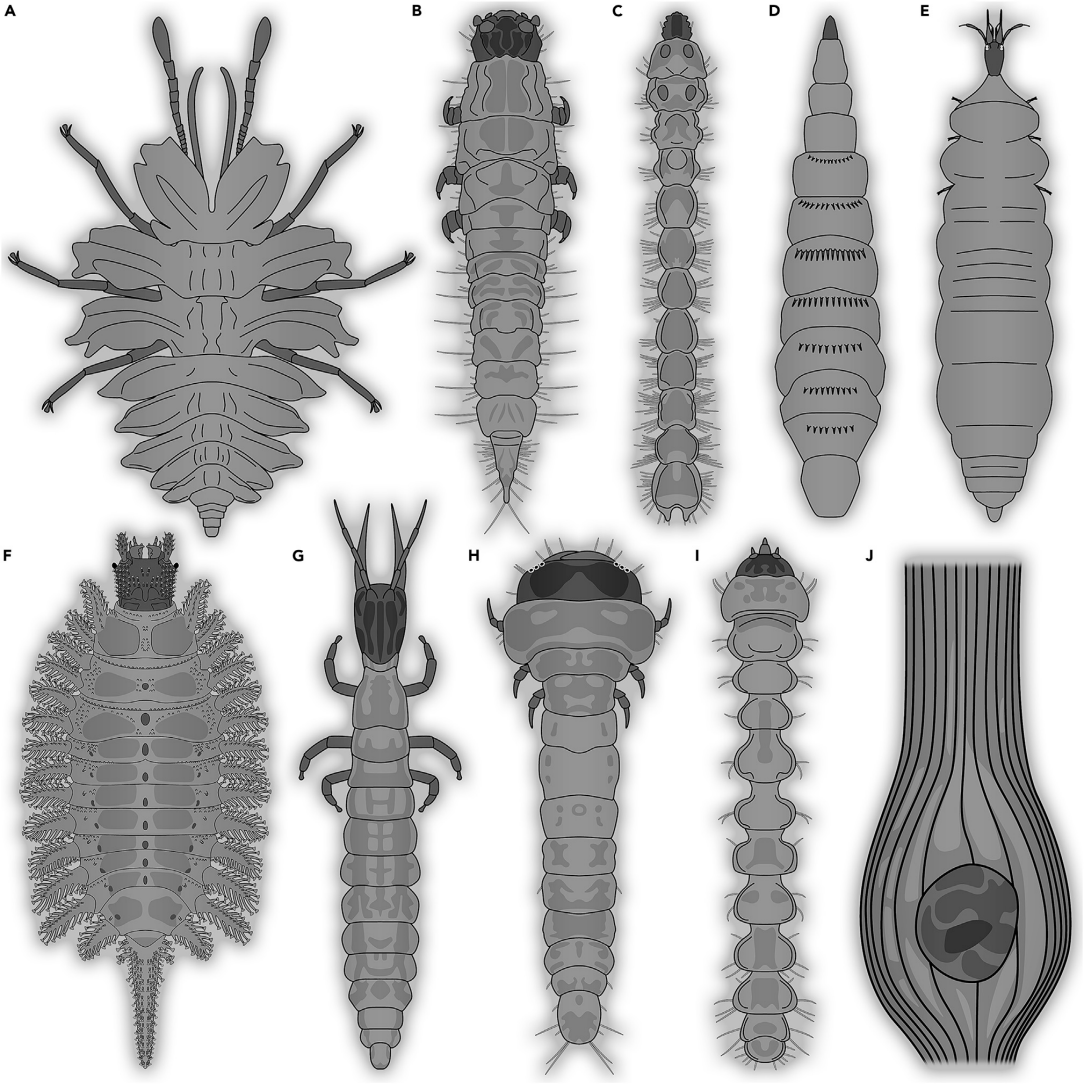
Various different types of defensive strategies in holometabolan larvae from Kachin amber or modern counterparts; drawings modified from various sources (A) Plant mimesis of a lacewing larva.^40^ (B–H) Larvae living inside wood. (B) Larva of a false flower beetle (Scraptiidae;^42^). (C) Larva of a false click beetle (Eucnemidae;^43^). (D) Larva of a soldier fly (Stratiomyomorpha;^44^). (E) Larva of a beaded lacewing (Berothidae;^45^). (F) Larva of a Texas beetle (Brachypsectridae;^46^). (G) Larva of a pleasing lacewing (Dilaridae;^47^). (H) Larva of a longhorn beetle (Cerambycidae;^48^). (I) Leaf-mining caterpillar.^49^ (J) Gall in a leaf from the Early Cretaceous.^50^

Imitating plants is a common strategy in different ingroups of Insecta.^37^^,^^38^^,^^39^ Yet, this strategy seems not so common in modern-day holometabolan larvae. A peculiar lacewing larva related to aphidlions from Kachin amber was originally interpreted to have mimicked a liverwort (Figure 3A; ^40^). Newer comparison shifted this interpretation slightly, as a mimesis of a lycophyte.^41^ Still the case demonstrates plant mimesis in the Kachin amber forest. Again, this type of mimesis may have been beneficial not only for predator detection/recognition avoidance, but also for detection/recognition avoidance by prey animals improving the hunting success.

Simpler forms of mimicry could involve a rather flat overall morphology and a darker color in order to make the animals rather undetectable on tree bark. There are some flat larvae that could well fall into this category, such as larvae of Brachypsectridae.^46^ Yet these larvae seem to prefer to additionally hide under pieces of bark or wood (see next point), hence it remains unclear if bark mimicry plays indeed a role here, but a combination of both seems at least possible.

#### Living inside a substrate: Wood

Living inside a specific substrate can be highly beneficial for a holometabolan larva. It may provide the larva with a specific food source, but it also makes detection by many predators unlikely; even if detection is possible, some substrates prohibit an easy access. Wood is an especially interesting substrate in this respect.

Some larvae living in softer, i.e., decaying wood are protected to a certain degree. Within Kachin amber, these are, for example, beetle larvae of the groups Scraptiidae (false flower beetles; Figure 3B; ^42^) and Eucnemidae (false click beetles; Figure 3C; ^43^) and very likely some dipteran larvae of the group Stratiomyomorpha (soldier flies and allies; Figure 3D; ^44^). These larvae help decaying wood, contribute to carbon cycling and are protected from detection and access in the rotten wood to a certain degree.

Also the predatory larvae of Brachypsectridae (Texas beetles) live associated with wood, either under loose bark or fallen off pieces of wood. More rarely they live in rock crevices.^51^ Also larvae of Brachypsectridae have been reported from Kachin amber (Figure 3F; ^46^^,^^52^). The larvae of Lymexylidae (ship-timber beetles) also live inside of decaying wood and feed on fungi that line the walls (e.g.,^53^). Adult representatives of Lymexylidae seems to be frequently found in different amber deposits, including Kachin, Baltic, Rovno, and Dominican amber (e.g.,^53^^,^^54^^,^^55^^,^^56^). However, fossil larvae are not known in the literature so far, but should have been abundant in the amber forests.

Yet, there are some possible specialized predators that still might have had access to larvae living within a substrate. Dinosaurs of the group Alvarezsauroidea, have been suggested to eat termites.^57^ Termites, especially the earlier representatives, likewise lived in wood; hence, these dinosaurs also have encountered the holometabolan larvae and most likely eaten them as well.

Some lacewing larvae also need to be discussed in this aspect. Many larvae of Berothidae in the modern fauna live inside termite nests.^58^^,^^59^ Most likely, they use the termites as a food source, but living inside an actively defended nest also provides some protective benefits (reduced detection, reduced access). Also fossil larvae of Berothidae (beaded lacewings) from Kachin amber show specializations related to living with termites (Figure 3E; ^45^), hence this strategy seems to have evolved 100 million years ago.

Other specialised predators that could access larvae in wood are other larvae. The larval stages of Dilaridae (pleasing lacewings) are known to live inside soil or wood to hunt other larvae.^60^ Also pleasing lacewing larvae are known from Kachin amber (Figure 3G; ^47^) and are likely to have employed a similar strategy. Also the extant larvae of Cleridae (checkered beetles) often lead the lifestyle of predators within wood tunnels dug by wood-boring larvae.^61^^,^^62^ The earliest fossil record of adult checkered beetles is known from the Middle Jurassic of China,^62^ and even though the wood tunnel–roaming larvae of Cleridae have not yet been recorded from Mesozoic ambers (only the parasitic larvae in Cretaceous amber, see later), it is well possible that the larvae with tunnel roaming habits already existed in this group in the Cretaceous.

Even more protective than rotten wood is hard wood. Here the access is further restricted. It seems unlikely that an animal with a wood-pecker-type strategy had evolved in the Cretaceous. Wood-peckers first occurred in the early Oligocene (around 30–34 mya).^63^ The group is assumed to have originated earlier (around 45 mya), but still 20 million years after the end of the Cretaceous.^48^ Hence the only threat to hard-wood-borer larvae would be other larvae hunting in the galleries, such as those of Dilaridae, also present in Kachin amber.^47^ Hard-wood-borer larvae of the beetle groups Buprestidae (jewel beetles) and possibly also Cerambycidae (longhorn beetles) occur in Kachin amber (Figure 3H; ^64^), apparently performing a quite modern strategy, being threatened only by very few highly specialized predators.

Despite the defensive advantages of living inside wood, major factors driving this lifestyle are more likely manifold. As pointed out, food sources are a major point; being able to feed on an omnipresent food source such as wood, or the fungi in the wood and, in consequence, on the many animals doing that was likely a major driving force for so many independent lineages entering this habitat. The advantages of defense as well as other factors such as a stable micro-climate may have been a minor additional factor, but may have well stabilized the positive selection forces.

#### Living inside a substrate: Leaves

While wood is a strong protective substrate, also other parts of larger plants can be used as a substrate to live in. One strategy, which is not strictly living inside a plant part, but very close to that, is building a shelter from leaves. This strategy is very common among butterfly caterpillars (e.g.,^65^^,^^66^^,^^67^^,^^68^^,^^69^). Leaf-mining larvae live indeed inside the rather limited space of a leaf. Leaf mines have been reported from Cretaceous leaves, attributed to jewel beetle larvae.^70^ Leaf-mining larvae are also known in different groups of Lepidoptera (moths), for example in Gracillariidae. While there are some examples of caterpillars that possess certain characters of Gracillariidae, many of these clearly possess no specializations for leaf mining.^49^^,^^71^ Fischer^72^ reported three caterpillars of *Phyllocnistis cretacea*, which show clear specializations for leaf mining (Figure 3I). The species was interpreted as an ingroup of Gracillariidae, yet Fischer^72^ already pointed out that the lack of well-developed prolegs in the specimens is unusual. Gauweiler et al.^49^ suggested that these larvae may as well represent leaf-mining caterpillars of a hymenopteran species.

**Figure 4:**
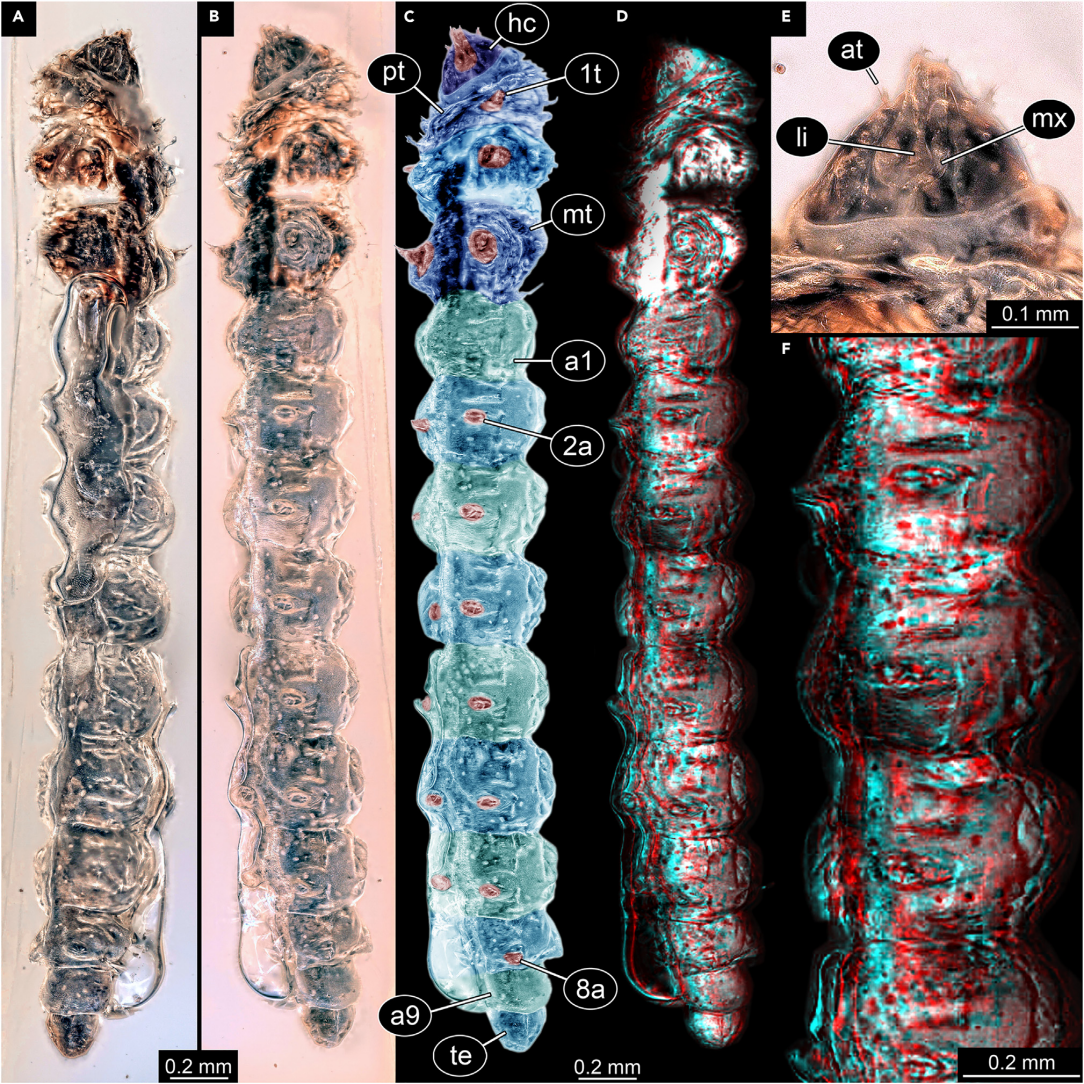
Leaf-mining caterpillar, BUB 4437 (A) Dorso-lateral view. (B) Ventro-lateral view. (C) Color-marked version of B. (D) Stereo anaglyph, use red-cyan glasses to view. (E) Close-up on head. (F) Stereo anaglyph of part of trunk with leglets, use red-cyan glasses to view. Abbreviations: at = antenna; a1 = abdomen segment 1; a9 = abdomen segment 9; hc = head capsule; li = labium; mt = metathorax; mx = maxilla; pt = prothorax; te = trunk end; 1t = appendage of trunk segment 1; 2a = appendage of abdomen segment 2 (proleg); 8a = appendage of abdomen segment 8 (proleg).

**Figure 5:**
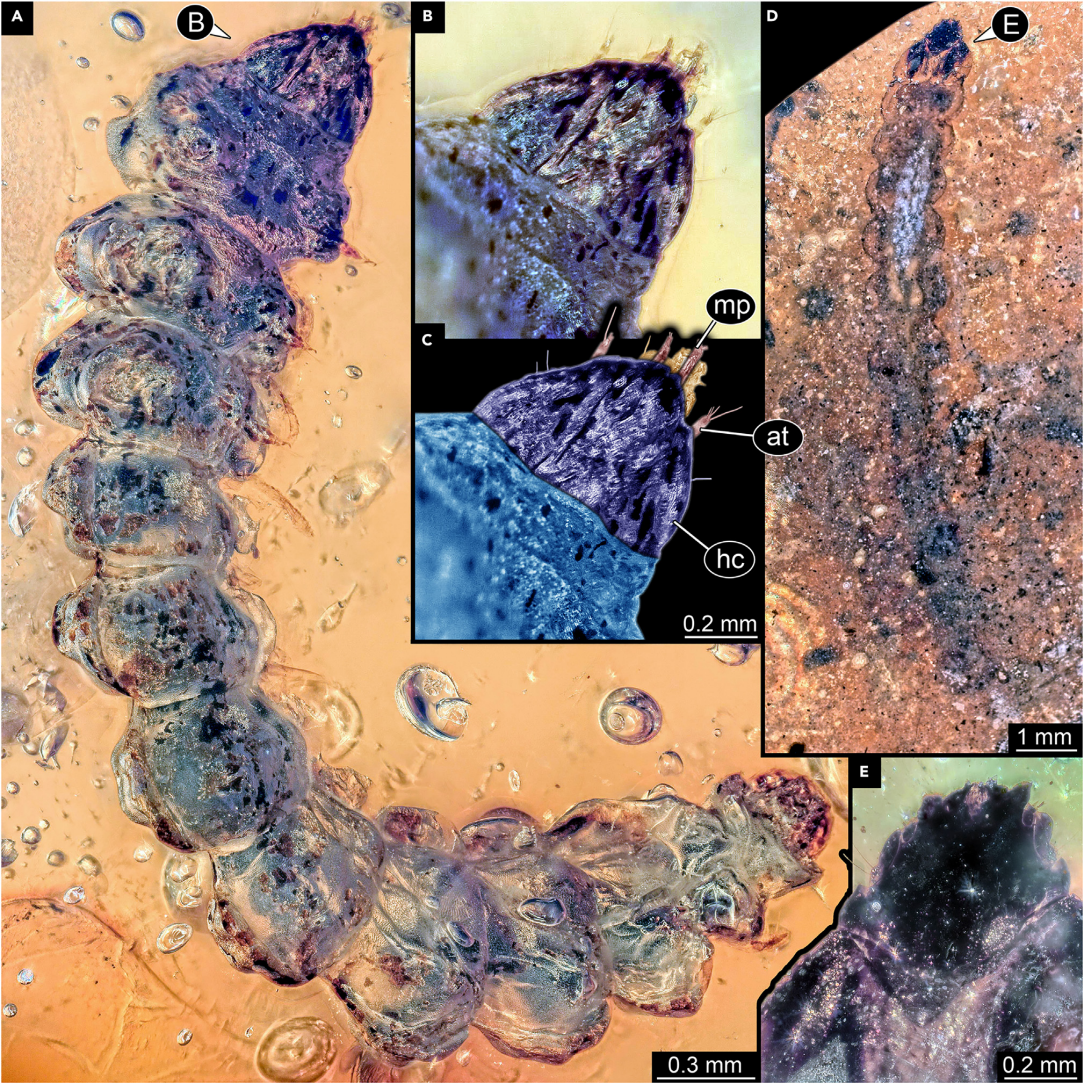
Leaf-mining caterpillars (A–C) PED 2303. (A) Ventral view. (B) Close-up on head. (C) Color-marked version of B. (D–E) PED 2546. (D) Dorsal(?) view. (E) Close-up on head. Abbreviations: at = antenna; hc = head capsule; mp = maxillary palp.

We here report new specimens resembling the three specimens from Fischer^72^ in many aspects (overall body shape, head shape, structure of tiny legs on thorax), also clearly showing specializations for leaf mining (Figures 4 and 5). One of the new specimens in addition reveals a detail not visible in the specimens of Fischer,^72^ but only when viewed in stereo-vision (Figures 4D and 4F): tiny prolegs on the abdomen, as suggested to be present by Fischer.^72^ In Gracillariidae, we would expect prolegs on abdomen segments 4–6, in other lepidopterans on 3–6; yet, the fossil has prolegs on abdomen segments 2–8. It is therefore highly unlikely that the new fossil represents a lepidopteran caterpillar, but more likely a hymenopteran one. We putatively suggest that, based on observable details, this new specimen is conspecific with the species *cretacea* described by Fischer^72^ (and the two not so well preserved larvae reported here), yet this would in consequence mean that this species would need to be removed from its suggested genus and transferred to Hymenoptera. A re-investigation of the three original specimens would be necessary for such an act. Still, we strongly support the interpretation of Fischer^72^ that the three larvae are leaf-mining caterpillars. We therefore have clear indications for leaf-mining larvae in the Kachin amber forest.

A very special way of using leaves (or also other parts of a plant) as protecting substrate is to modify the plant metabolism to induce tumor-like outgrows or galls. These galls are induced, for example, by representatives of Hemiptera or Thysanoptera, but mainly by holometabolans to protect their offspring. Examples include gall midges (Cecidomyiidae) and several hymenopterans such as gall wasps (Cynipidae^73^). Fossil remains of galls have been frequently reported (Figure 3J; e.g.; ^50^ recently summarized in^74^). The oldest fossil galls discussed to have been produced by holometabolans came from the Carboniferous Mattoon Formation of the Illinois Basin.^75^ Such galls have not been directly observed in Kachin amber, but are highly likely to have been present there.

Similar to living in wood, the main selective forces leading to the evolution of living inside leaves may have been related to food. Yet, quite some other advantages such as protection and micro-climate will have been beneficial for the animals also in this case.

#### Living inside a substrate: Soil

Living in the ground, or better the soil, is also an effective strategy to reduce detection and access. Many modern holometabolan larvae perform such a lifestyle. Often, but not always, this lifestyle is related to a scarabaeiform morphology, i.e., a grub-type appearance. So far, we have no cases of a strict grub-like appearance in Kachin amber, neither from beetle larvae nor from other groups such as Ithonidae (moth lacewings).

**Figure 6:**
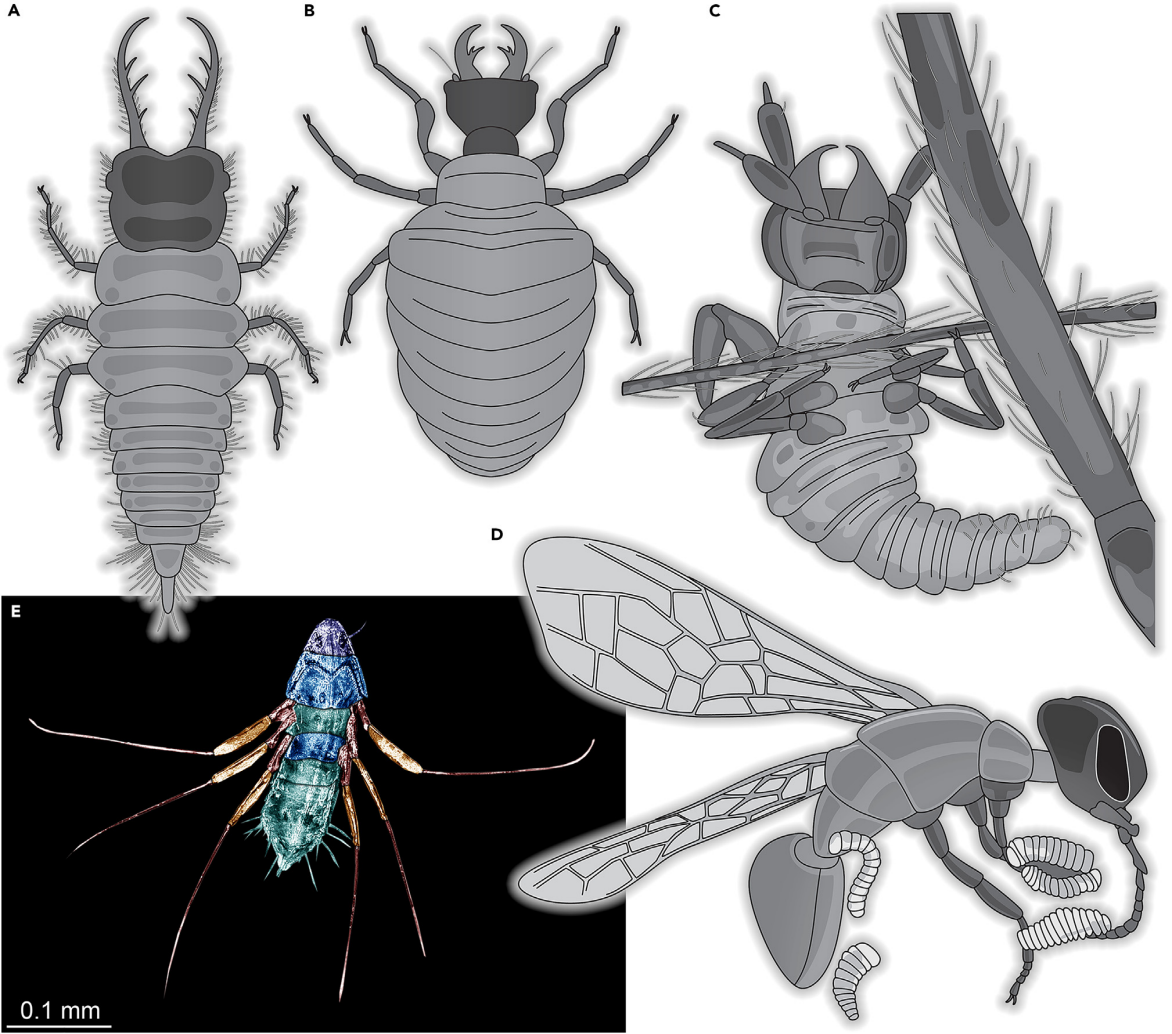
Various different types of defensive strategies in holometabolan larvae from Kachin amber; drawings modified from various sources (A–B) Digging larvae. (A) Antlion larva (Myrmeleontidae) with digging setae.^75^ (B) Presumed larva of spoon-winged lacewings (Nemopterinae). (C–E) Parasites in the wide sense. (C) Larva of mantis lacewings (Mantispidae) climbing onto a spider leg.^77^ (D) Triungulin larvae of checker beetles (Cleridae) closely associated to an early bee.^78^ (E) Triungulin larva of wedge-shaped beetles (Ripiphoridae), color-marked (SMNS BU 60_12).

Still, there are some larvae that have indications for a digging lifestyle. Badano et al.^7^ reported many peculiar lacewing larvae, among them a sole representative of Myrmeleontidae (antlions), which possesses some digging setae (Figure 6A). As for camouflaging in lacewings, the digging behavior of antlion larvae is mostly an aggressive feature, in order to optimize hunting success. Since there was also predation pressure on lacewing larvae back in the Kachin amber forest,^76^ avoiding predator detection seems to have been a likely aspect of the digging behavior of these early antlion larvae as well.

Other larvae were supposed to be related to groups that perform flat digging^79^ or also deep digging (Figure 6B; ^77^). Yet, these larvae possibly performed also different ecological roles and were not necessarily digging.^77^^,^^79^ However, it cannot be fully excluded that they dug at least to a certain degree.

As for living inside wood and living inside leaves, living inside the soil likewise was driven by food availability. Yet, here not necessarily the substrate itself acts as the food source, but the many organisms in it or, in the cases of lacewings, above it. Also, here defense and micro-climate may be understood as additional factors that were beneficial.

#### Parasitism and related

Besides soil and plants, also animals can be an effective protective substrate to live in or on. Although the latter may be less effective concerning detection, the often larger host is expected to defend itself against predators more effectively than the often much smaller parasitic larva. An example is a small larva of Mantispidae (mantis lacewings), which was preserved in amber in the moment of climbing onto its host, a spider (Figure 6C; ^78^). Later in ontogeny, modern mantis lacewing larvae wait until the spider has produced an egg sac, enter the egg sac, and live protected in there. We can assume that the fossil larva would have done the same. There are also other similar strategies in modern lacewing larvae, for example, entering nests of eusocial insects, feeding on eggs and larvae in the nest, and being protected in there. Quantitative analysis indicates a diversification of the morphology of mantis lacewing larvae after the Cretaceous, also indicating a diversification of ecologies.^47^ It is therefore well possible that the strategy to live in nests of eusocial insects evolved only after the Cretaceous.

Poinar and Brown^80^ reported triungulin larvae of the group Cleridae (checker beetles) closely associated to an early bee (Figure 6D). In modern checker beetles, some triungulin larvae mount bees, are carried to the nest, and live inside the nest, quite similar to some modern mantis lacewing larvae. It appears that this strategy was already present in the Kachin amber forest.

**Figure 7:**
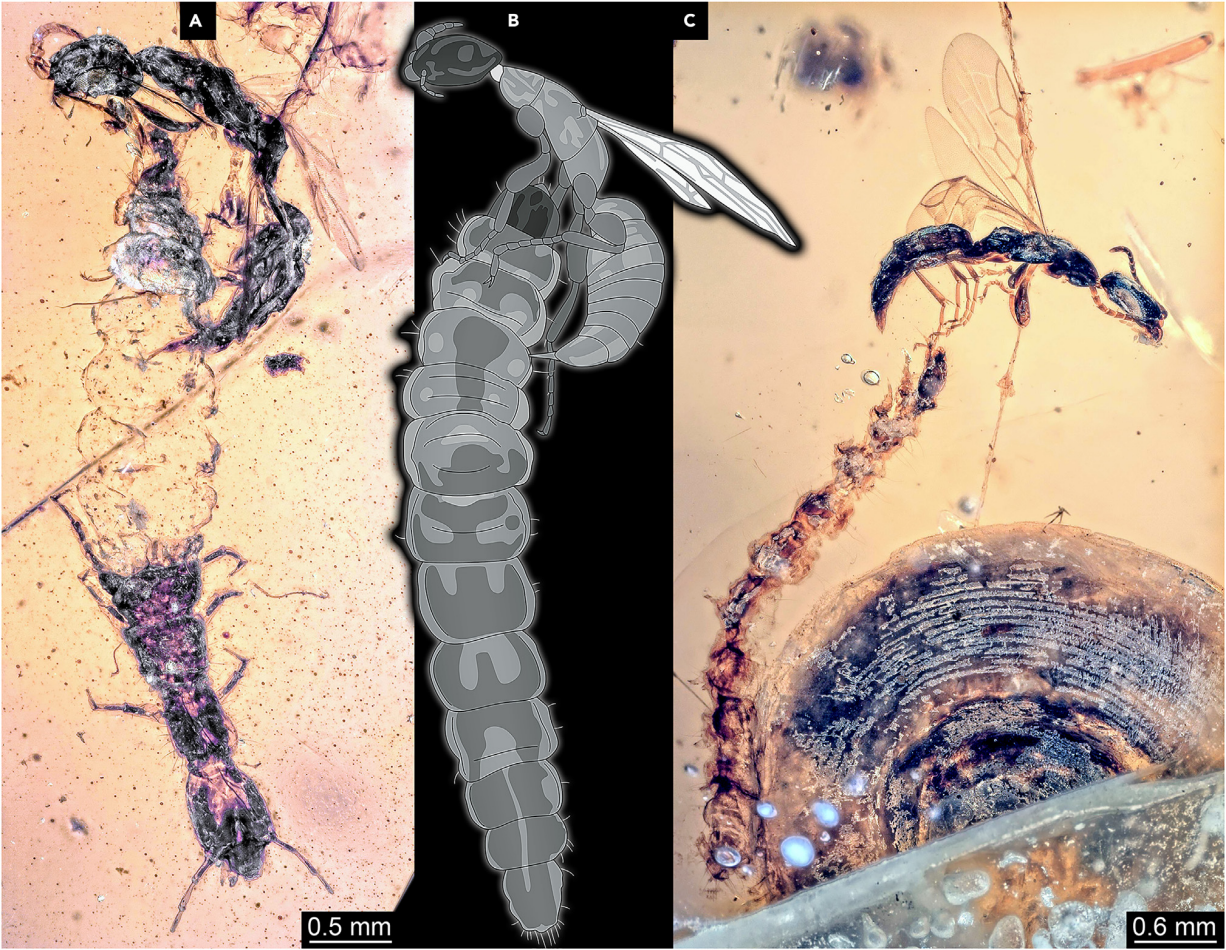
Flat wasps (Bethylidae) trapped in Kachin amber while stinging into host larvae for oviposition (A) Snakefly larva as host, PED 2351. (B) Beetle larva as host.^81^ (C) Lepidopteran caterpillar as host, PED 2575.

Although the modern fauna is full of parasites, examples preserved in amber are in fact relatively rare. Most cases are restricted to the mere presence of adults for which we know that in the modern fauna their larvae are parasitic. A single wasp of the group Bethylidae (flat wasps) is so far known from Kachin amber preserved while stinging into a possible host for its offspring (Figure 7B; ^81^), indicating that the wasps offspring lived parasitic on a larger beetle larva. New specimens indicate that not only beetle larvae were possible hosts, but also snakefly larvae (Figure 7A) and lepidopteran caterpillars (Figure 7C).

Later larval stages of Strepsiptera and Ripiphoridae live as endoparasites, hence are protected within their host against possible predators. The triungulin (first stage) larvae of both groups, which search for a host, are known in Kachin amber (Figure 6E). These fossils indicate that they might have performed a lifestyle similar to that of the modern forms already in the Cretaceous.^82^^,^^83^^,^^84^

Parasitism seems very similar to living inside wood and leaves, both can be understood as plant parasitism.^64^ Therefore, also here the same selective forces should apply, opening a new type of food source, with protection and a stable micro-climate as additional benefits.

### Passive defense strategies

#### Defensive structures: Spines and hairs

**Figure 8:**
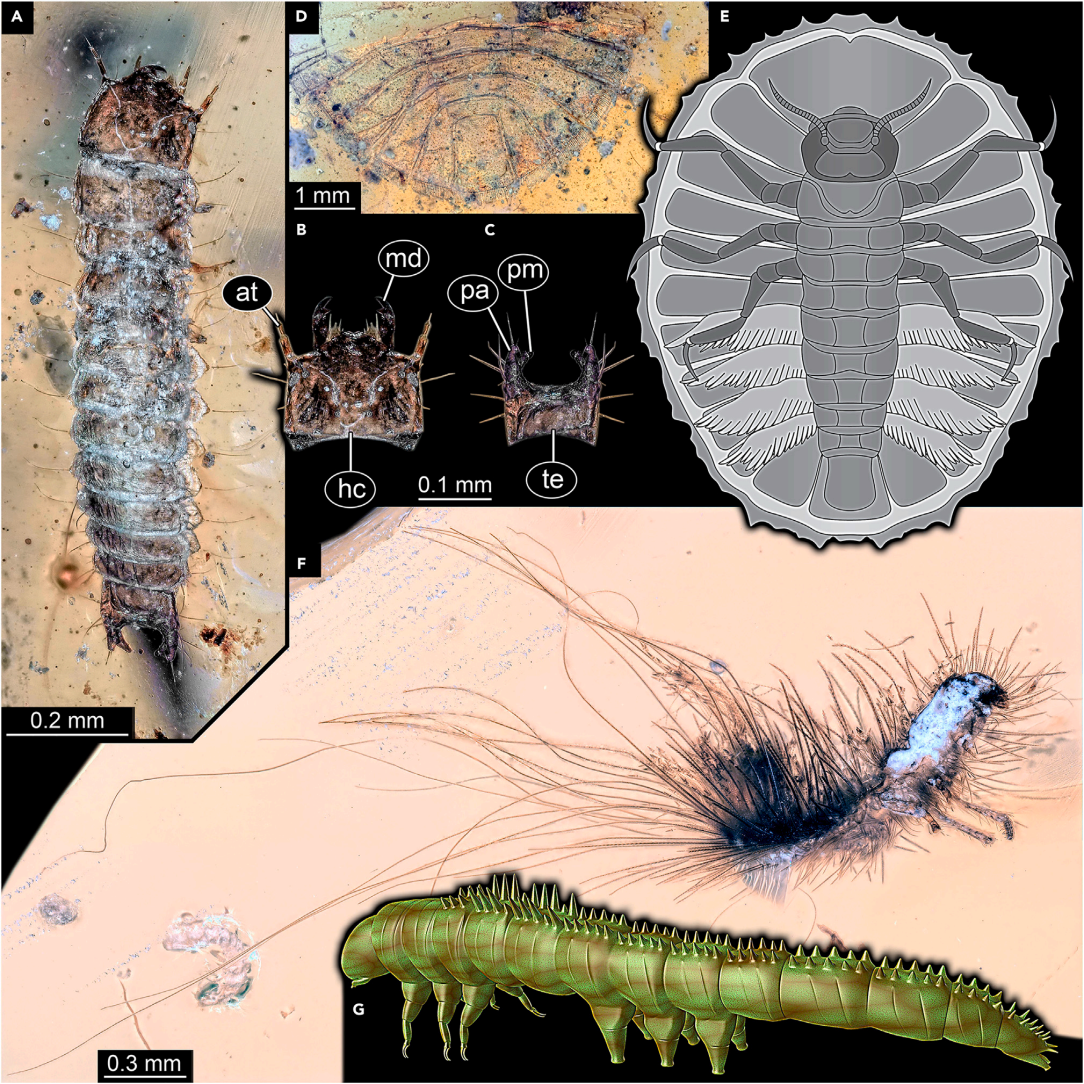
Various different types of defensive strategies in holometabolan larvae from Kachin amber (A–C) Self-mimicry of a beetle larva, PED 2120, compare B and C. (A) Overview. (B) Symmetrized head. (C) Symmetrized trunk end. (D–E) Water pennies, larvae of Psephenidae. (D) Incomplete specimen, posterior trunk end, PED 2240. (E) Ventral view on complete specimen.^87^ (F) Larva of skin beetles (Dermestidae) with long hairs, PED 1369. (G) Reconstruction of lepidopteran caterpillar with stout spines dorsally.^70^ Abbreviations: at = antenna; hc = head capsule; md = mandible; pa = pseudo-antenna; pm = pseudo-mandible; te = trunk end.

Among the most obvious defensive structures are spines and hairs, as exemplified par excellence by many modern-day caterpillars. Astonishingly, most Cretaceous caterpillars are of the “naked” type. An exception is a single small lepidopteran caterpillar with numerous stout spines on the dorsal side (Figure 8G; ^71^). In younger ambers, also more complex structures have been reported, such as poison bearing spines^85^; also long hairs are known as defensive structures of caterpillars.^86^ So far, such specializations are not known from Cretaceous caterpillars.

Among beetles, the larvae of Dermestidae are also covered in long, dense hairs. These larvae are known for being scavengers that feed on dry organic material such as wood or organic remnants on bones. Fossils of conspicuous larvae of Dermestidae have been reported, e.g., in Dominican, Baltic, Sakhalinian, and Kachin amber (Figure 8F; ^53^^,^^88^^,^^89^^,^^90^^,^^91^).

#### Defensive morphology: Unusual shape or size

A predator has to get a grip on its prey. If the prey is very flat, it can prove pretty challenging to lift the prey item off the substrate. Larvae of Psephenidae (water penny beetles) have very flat bodies, with setae along the outer rim, allowing a close attachment to their substrate under water (Figures 8D and 8E). The combination of flat body and effective attachment is a specialization to faster-running waters, but also provides a certain protection against predators.^87^ Fossil larvae of Psephenidae are known in Kachin amber (Figure 8E; ^92^), possessing the same specialized morphology as their modern counterparts.

Prey size can affect the success of a predation attempt. Yet, opposed to other animals, holometabolan larvae tend to be consumed more often with increasing size,^93^^,^^94^ likely due to increased detectability.^95^ Most birds and predatory representatives among Euarthropoda have tools to deal with larger prey items.^96^ Increasing size only seems to have a positive survival effect on holometabolan larvae when combined with other defensive structures.^97^ This could be a possible explanation why all currently known caterpillars from the Mesozoic are rather small, as almost none of them (with the exception of one) has spines or thick long hairs.

Enrolling or curling up into a ball, only exposing well-sclerotized plates to the exterior, is an effective defensive strategy that is employed by numerous different terrestrial and aquatic representatives of Euarthropoda.^98^^,^^99^ Among holometabolans, the strategy is rarer, but is known, for example, for certain larvae of the beetle group Scydmaeninae.^100^^,^^101^ While a larva of Scydmaeninae is known from Kachin amber,^102^ it does not possess a morphology indicative of abilities to enroll or curl up. This specific strategy does not seem to be present in holometabolan larvae of the Kachin amber forest to present knowledge.

#### Protective cases

Larvae of different holometabolans use various materials to build protective cases, including beetles (e.g.,^103^^,^^104^), moths (e.g.,^2^^,^^105^^,^^106^^,^^107^^,^^108^), and especially caddisflies (e.g.,^109^^,^^110^). Such protective cases are well known from younger ambers (e.g.,^86^), but have so far not been reported from Kachin amber.

#### Aposematism

Aposematism is a well-known phenomenon of protective coloration, most famous in adult wasps. Aposematism in larvae is much rarer. A non-holometabolan immature orthopteran from Kachin amber has been recently reported to exhibit aposematism,^20^ but so far no holometabolan larva with such a kind of coloration is known.

#### Mimicry

While there are different types of mimicry, in the here discussed context defensive mimicry (Müller’s and Bates’ mimicry) is of importance. Yet, as there are, for example, no clear cases of aposematism in the group in focus, there is no target animal to be mimicked; therefore, it should not be surprising that we do not have a clear case of these types of mimicry among the holometabolan larvae from Kachin amber.

Mimicry usually refers to an organism that mimics the appearance (optically, chemically, or other) of another organism, and it comes in a variety of types.^111^ Yet, there is one exception, a type of mimicry in which only a single individual is involved: self-mimicry, which describes cases in which a part of an organism resembles another part of the same individual.^112^^,^^113^^,^^114^^,^^115^^,^^116^ A common case of self-mimicry in animals is that the posterior end resembles the head, providing the impression that the body end is the head (in snakes^112^^,^^113^; in caterpillars^117^).

The use of the term and the phenomenon of self-mimicry is unfortunately not uniform. Some authors have used the term ‘automimicry’ to refer to cases in which one part of the body resembles another part of the body.^117^^,^^118^^,^^119^^,^^120^ Yet, more often the term automimicry has been used to refer to cases in which some individuals of a species (or a population) mimic other individuals with different properties.^114^^,^^115^^,^^120^^,^^121^ An example is represented by individuals of a species that are dangerous (e.g., poisonous) and use aposematic (warning) coloring, while others are using aposematic coloring without being dangerous (e.g.,^116^^,^^122^^,^^123^^,^^124^^,^^125^), hence representing a special case of Batesian mimicry.^120^ This is advantageous for the mimicking animal as it does not need to spend energy on producing the poison.

Also the term self-mimicry has been applied differently, for example, to cases in which different types of patterns of an organism resemble each other, e.g., pigment pattern and bioluminescence pattern in gelatinous animals,^126^ or a case in which a spider models its prey items in a way to resemble itself.^127^ Also, cases in which one part of an organism resembles another one has been termed 'self-crypsis'.^128^ As the majority of sources seems to use self-mimicry for cases of resemblance of different parts of a single organism, the term is used in this way here.

Some beetle larvae possess trunk ends that in dorsal view largely match the shape of the head capsule (Figures 8A–8C). As the body of such larvae is usually not tapering or only slightly, this makes the evaluation tricky, which part is the front. In addition, the trunk ends of such larvae often have processes which resembles the spread-open mandibles and the antennae. In combination, the morphology is a good candidate for a case of effective self-mimicry.

### Active defense strategies

#### Biting

Many holometabolan larvae possess prominent mouth parts and are capable of biting a predator; in extreme cases and eusocial systems, defensive biting may lead to suicidal biting.^129^ Also many larvae known from Kachin amber have very prominent mouth parts, especially mandibles, which may have allowed defensive biting. Yet, we have no additional data substantiating this assumption.

#### Group defense

**Figure 9:**
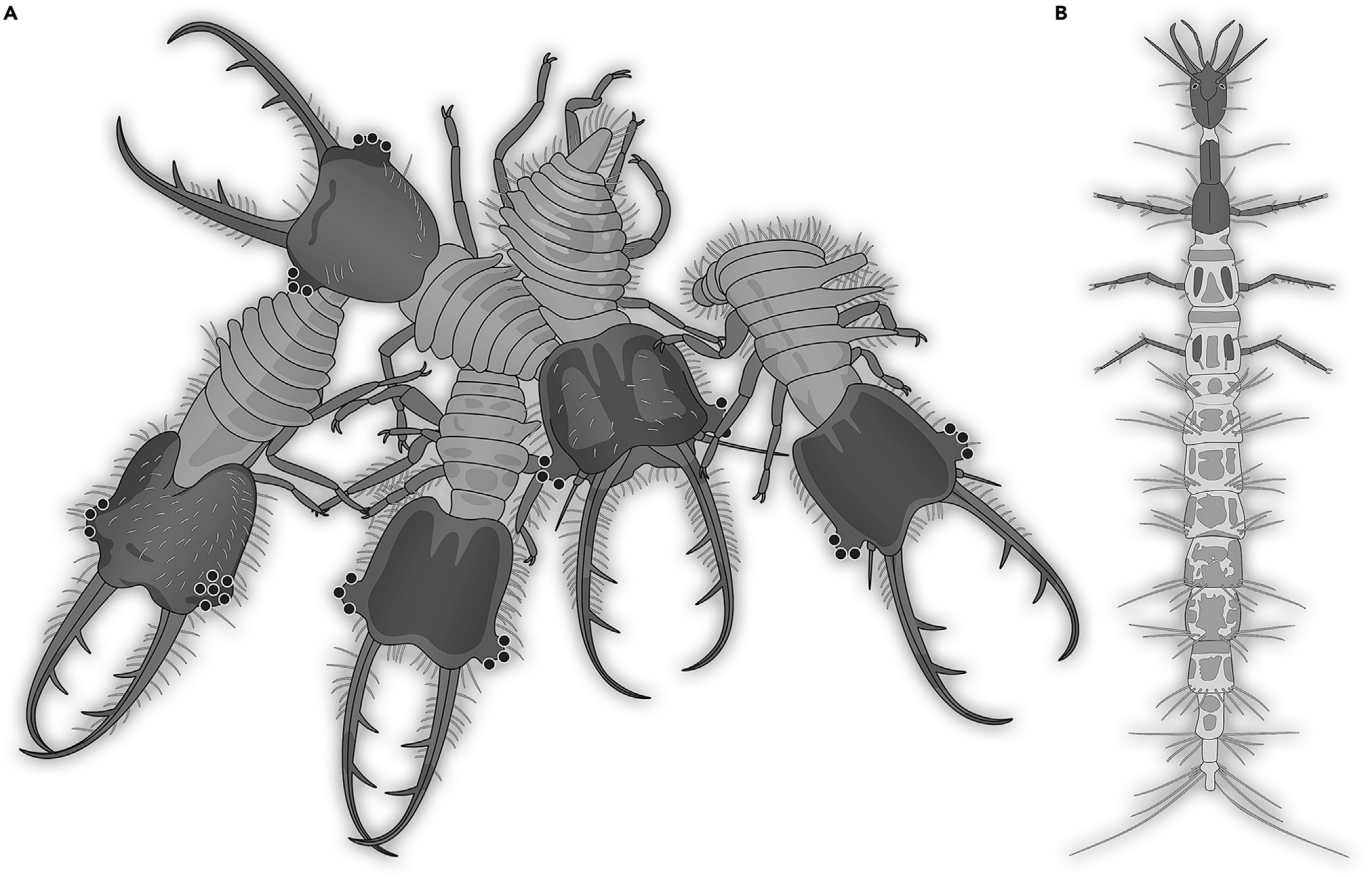
Various different types of defensive strategies in holometabolan larvae from Kachin amber or modern counterparts (A) Possible case of group defense in owllion larvae.^36^ (B) Larva of a dragon lacewing (Nevrorthidae) with very flexible body.^132^

Many holometabolan larvae occur in groups and can perform coordinated group defense. A possible case of such a group of larvae has been reported for owllion larvae from Kachin amber (Figure 9A; ^36^). These animals are unlikely to be directly related to modern owllions performing this type of defense,^130^^,^^131^ hence this specific behavior must have evolved at least two times independently in lacewings.

Group defense can also be part of brood care. While this is commonly a behavior of the adults, larvae benefit from it. This behavior is well known in many extant (eu-)social holometabolan species, such as bees, ants and other wasps. There is no direct indication for such a behavior in Kachin amber, but as eusocial forms are known (e.g.,^133^), we can assume such a kind of behavior. Also modern non-eusocial species have sophisticated brood care (e.g.,^134^), yet it is difficult to substantiate such behavior in fossils.^36^

#### Evasion

When being threatened by a predator, an animal can also try to evade it. Jumping is a common mechanism to do so, well known in many non-holometabolans (springtails, jumping bristletails, orthopterans, treehoppers, planthoppers) and some adult holometabolans (fleas), but rarely in larvae.^135^ So far, we have no case of a holometabolan larva in Kachin amber possessing indications of such a behavior.

Some extant caterpillars also employ a behavior called ballooning. They can release a silk strand as they drop off a leaf to escape a predator. They rappel down and climb back up onto another leaf. There is a fossil caterpillar in Baltic amber that has been identified as representative of Geometridae,^132^ a group that has multiple extant ingroups showing this kind of defensive behavior. The fossil also preserves remains of the silk. Yet, so far no comparable fossil has been reported in Cretaceous ambers.

Nevertheless, other types of evasion are also possible. Larvae of Nevrorthidae (dragon lacewings) have strongly elongated bodies with additional intra-segmental joints (Figure 9B; ^136^), likely enabling them to move between pebbles or alike. While this may be beneficial for hunting prey, it also enables them to hide between the pebbles when threatened. Larvae of Nevrorthidae with very similar morphologies are known in Kachin amber.^136^^,^^137^^,^^138^

### Conclusions and outlook

Larvae from the extant fauna are less often present in the literature than the adults, a side effect of taxonomy focusing on adult features. The imbalance of larvae vs. adults is even more expressed in the fossil record. Simply checking the literature might provide the impression that holometabolan larvae are rare, even in amber, but in fact, they are rather common. However, a taxonomic treatment is often challenging (e.g.,^51^^,^^139^), simply inconclusive^140^^,^^141^^,^^142^ or causing opposing views (e.g.,^143^ ^vs^ ^82^^,^^144^ ^vs^ ^145^ ^vs^ ^146^). Still, holometabolan larvae represent a major component of the modern food web and appear to have been similarly so in the past. However, there seems to have been no focused search for such cases, and the wealth of examples as lain out here is just an indication that there must have been many more strategies so far not recognized in the Kachin amber forest.

Reconstructing such food webs is still very challenging, as there are only very few cases of direct interactions of predator and prey (e.g.,^76^^,^^147^) or even simple co-occurrences of prey and possible food items.^148^ Therefore, more indirect types of indicators need to be used, and more fossils with indications of certain strategies have to be included in future studies. The anti-predator strategies of holometabolan larvae preserved in about 100-million-year-old Kachin amber are very diverse, even much more diverse than recently summarized by Xu et al.^20^^,^^21^

Also some of the strategies sub-summarized here need to be understood in a more complex frame. Especially the different cases of detection avoidance in which the immatures live inside a substrate (wood, leaves, soil, living animals) led to a secured new food source and a stable micro-climate, but, due to the detection avoidance, also an effective defense strategy.

There are predators^149^^,^^150^ and parasitoids^151^^,^^152^^,^^153^ specialized in locating these larvae in their substrates. Yet, these predators and parasitoids are highly specialized, and in fact only few lineages managed to exploit the specialized larvae living inside the different substrates as a food source. This demonstrates that the principle strategy to live inside a substrate is an effective defense strategy that demands predators (and parasitoids) to overcome this defense, but to evolve discrete specializations.

There seem still to be several defense strategies known from the modern fauna or younger ambers that have not been encountered in Kachin amber, yet the findings from this Lagerstätte are still increasing, and it still does not appear that we have reached a saturation point, i.e., it does not seem that the reports of new findings will slow down soon. Kachin amber therefore is a currently unique window into the past, providing us with an extreme wealth of biological information from a now extinct ecosystem.

A point to be emphasized is that although we see many anti-predator strategies we know from modern faunal components, they are not necessarily performed by directly related forms. Instead, it appears that many strategies have evolved repetitively independently due to convergent evolution. Such a pattern indicates that there are especially successful strategies that have been employed by many lineages, also many modern lineages, and that evolved repetitively. In contrast to that, there are also strategies that are rarer, especially among holometabolans. Such patterns have the potential to reveal more details about selective pressures and costs of specific strategies, but require a large database, one that we have here started to build.

### Limitations of the study

The study is based on currently known examples in the context of anti-predator strategies from the literature and 13 newly discovered specimens. Many specific strategies in this context that are observed in groups living today are not represented in the fossil record up to now. The present study therefore provides a minimum range of anti-predation strategies for Cretaceous holometabolan larvae. There was probably a variety of other strategies, but examples of these have not yet been discovered.

## STAR★Methods

### Key resources table

**Table:** 

REAGENT or RESOURCE	SOURCE	IDENTIFIER
**Software and algorithms**		

Post-processing of all images and color markings were performed with Adobe Photoshop CS2	Adobe Inc.	RRID:SCR_014199; URL: https://www.adobe.com/products/photoshop.html
Drawings were performed in Adobe Illustrator CS2	Adobe Inc.	RRID:SCR_010279; https://www.adobe.com/products/illustrator.html

### Resource availability

#### Lead contact

Further requests concerning the investigated material should be directed to and will be answered by the lead contact, Marie K. Hörnig (marie.hoernig@palaeo-evo-devo.info or marie.hoernig@med.uni-rostock.de).

#### Materials availability

Most investigated specimens originate from literature data. Additionally, specimens were legally purchased from the internet platform ebay.com from the traders burmite-miner and burmitefossil. The specimens are now part of the Palaeo-Evo-Devo Research Group Collection of Arthropods, Ludwig-Maximilians-Universität München, Germany (PED 0377, PED 0642, PED 1369, PED 1986, PED 2120, PED 2240, PED 2303, PED 2351, PED 2546, PED 2575). Other specimens come from the collection of one of the authors, PM (BUB 3724, BUB 4437) and from the Staatliches Museum für Naturkunde Stuttgart (SMNS BU 60 12).

#### Data and code availability

• All data reported in this paper will be shared by the lead contact upon request.
• This paper does not report original code.
• Any additional information required to reanalyze the data reported in this paper is available from the lead contact upon request.

### Method details

The documentation of the specimens at hand was performed on a Keyence VHX-6000 digital microscope under different types of illumination (white or black background; low-angle ring illumination or co-axial cross-polarised illumination^154^). Each image was recorded as a composite image of several adjacent image stacks, which were calculated into a large and fully sharp panorama by the built-in software. Post-processing of images and colour markings were performed with Adobe Photoshop CS2. Drawings of specimens from the literature were performed with Adobe Illustrator CS2.
